# Estimating the size of hidden populations from register data

**DOI:** 10.1186/1471-2288-14-58

**Published:** 2014-04-27

**Authors:** Anders Ledberg, Peter Wennberg

**Affiliations:** 1 Centre for Social Research on Alcohol and Drugs, SoRAD, Stockholm University, SE-10691 Stockholm, Sweden

**Keywords:** Prevalence, Hidden population, Capture-recapture, Truncated Poisson, Opiates, Heroin, Mortality

## Abstract

**Background:**

Prevalence estimates of drug use, or of its consequences, are considered important in many contexts and may have substantial influence over public policy. However, it is rarely possible to simply count the relevant individuals, in particular when the defining characteristics might be illegal, as in the drug use case. Consequently methods are needed to estimate the size of such partly ‘hidden’ populations, and many such methods have been developed and used within epidemiology including studies of alcohol and drug use. Here we introduce a method appropriate for estimating the size of human populations given a single source of data, for example entries in a health-care registry.

**Methods:**

The setup is the following: during a fixed time-period, e.g. a year, individuals belonging to the target population have a non-zero probability of being “registered”. Each individual might be registered multiple times and the time-points of the registrations are recorded. Assuming that the population is closed and that the probability of being registered at least once is constant, we derive a family of maximum likelihood (ML) estimators of total population size. We study the ML estimator using Monte Carlo simulations and delimit the range of cases where it is useful. In particular we investigate the effect of making the population heterogeneous with respect to probability of being registered.

**Results:**

The new estimator is asymptotically unbiased and we show that high precision estimates can be obtained for samples covering as little as 25% of the total population size. However, if the total population size is small (say in the order of 500) a larger fraction needs to be sampled to achieve reliable estimates. Further we show that the estimator give reliable estimates even when individuals differ in the probability of being registered. We also compare the ML estimator to an estimator known as Chao’s estimator and show that the latter can have a substantial bias when applied to epidemiological data.

**Conclusions:**

The population size estimator suggested herein complements existing methods and is less sensitive to certain types of dependencies typical in epidemiological data.

## Background

We consider the problem of estimating the size of an incompletely sampled population. That is, given “information” on some individuals from a more less well-defined population we want to estimate the total number of individuals in this population. A typical example could be to estimate the total prevalence of “problem drug use” from known cases in health-care or judiciary records. This is a question of some priority both at national (e.g. [1,2]) and international (e.g. [3]) levels.

There are two related approaches that have been previously applied to similar problems given epidemiological data, including data on alcohol- and drug misuse (e.g. [4,5]). In the first approach individual data from a number of different sources are collected and matched. Prevalence estimates are then formed using so-called capture-recapture techniques (e.g. [6]). The types of data sources used obviously depend on the target population and might include records from: health care, treatment programs, needle exchange program, police, and prison system (e.g. [7,8]).

In the second approach, data from only one source is used and a probability model of the registration frequencies is assumed. This probability model is typically a zero-truncated distribution, for example truncated Poisson. The zero frequency is estimated from data and an estimate of the population size is then obtained [9-12].

Both these approaches rely on a number of assumptions that may or may not hold in real situations (e.g. [4,13]). The approach using multiple sources suffer from a non-clear definition of the target population (e.g. do needle exchange program and police records for drug offenses really target the same population?) as well as general problems of dependencies between samples. The second approach, relying on a single source, avoids the first problem but might give strongly biased estimates unless a reasonable model of the frequency distribution is chosen.

Here we suggest a novel approach to population size estimation from a single source of data. This approach is tailored to epidemiological data and relies on a minimum of assumptions. We assume that all individuals in the target population have a non-zero probability of being registered during a given time interval. By using only the time-points of first contact with the registry we avoid effects of previous registrations. Given this setup, it is straight forward to derive the maximum likelihood (ML) estimator of the population size (*N*). We note that the resulting approach is formally identical to the capture-and-remove approach previously used in populationbiology (cf. [14-16]).

We evaluate the ML estimator of *N* using extensive Monte Carlo simulations and further compare its performance to another estimator known as Chao’s estimator [17]. The two estimators are also applied to health care registry data in a case where the true *N* is known.

## Methods

### Outline and notation

**Figure 1:**
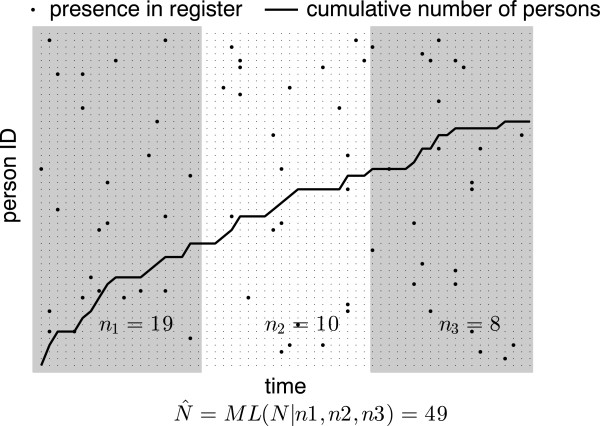
**Illustration of estimation method.** Raster represents person IDs (total 50 persons, *N*=50) vs time (60 time-points). Black dots illustrates presence in the register. Here a fixed registration probability of 0.025 was used (i.e. *π*=0.025). Time is divided into three epochs (i.e. *M*=3) of equal duration (indicated by the shading). In each epoch the number of previously unregistered persons are counted: first epoch *n*_1_=19, second *n*_2_=10, and third *n*_3_=8. These observed counts are used to estimate the total number of persons in the population. Estimation is done by maximizing the likelihood of *N* and *p* given the data. For the example the data in the figure the estimate of *N* equals 49.

We consider the following situation (see Figure 1 for illustration): The target population (consisting of *N* individuals) is followed over time during a time interval of total duration *T*. At each time-point a given individual may or may not be registered. We use ‘registered’ in a general sense here, it can for example refer to an instance of care at a medical facility; an arrest by the police; the purchase of a medically prescribed drug, etc. We assume that each individual in the target population, if not previously registered, has a fixed probability *π* of being registered at each time-point. We will refer to *π* as registration probability in the following. A registration (independent of the nature of the registry), must contain an identifier of the person (PIN) and the time-point of registration. We note that there might be a delay between the occurrence of the event we would like to track, and the time point of registration of this event. Consider for example an attempt to estimate the prevalence of a certain disease in the population. If health care records are used to estimate this, there might be a delay between the time of acquiring the disease and the first contact with the health care system. We will assume that such delays, if they occur, do not introduce systematic errors in the analysis and hence can be ignored.

Next we will detail how such register data can be used to estimate the total size *N* of the population. First we will describe the novel ML approach and then an approach that has been previously used with data of thistype.

### Maximum likelihood estimation of *N*

Here we describe how the data in the register can be used to estimate *N* using ML (see Figure 1 for illustration). First the observation interval is divided into *M* non-overlapping epochs with equal duration *d*. That is, the *j*-th epoch, *I*_*j*_ is given by *I*_*j*_=[*t*_0_+*d*(*j*−1),*t*_0_+*dj*) where *t*_0_ denotes the time point when the observations start and *j*=1,2,…,*M*. We will assume that time is discrete and that there are *d* opportunities to be registered in an interval of duration *d*. For each of the *M* epochs we count the number of new persons being registered in that epoch. That is, we count the persons registered during epoch *I*_*j*_ that were not registered in any of the previous epochs. These numbers will be called *n*_1_,*n*_2_,…,*n*_*M*_ and are used in the following to represent both random variables and samples of these random variables. It is *n*_1_,*n*_2_,…,*n*_*M*_ that constitute the data we will use to estimate the total number of persons in the population (i.e. *N*). The number of intervals *M*>1 is a parameter in the suggested method and we will study how the estimate of *N* depend on *M* below.

We proceed by deriving the ML estimator under rather idealized conditions (a closed and homogeneous population) but will later study numerically how the estimator performs when we make the population heterogeneous. Assuming that the target population does not change in the time window during which we make the observations, and that each member of the population has the same registration probability, *π*, it is straight-forward to write down the probability distribution of the sample *n*_1_,*n*_2_,…,*n*_*M*_. Indeed, the number of persons registered in the first epoch, i.e. *n*_1_, is distributed as a Binomial random variable with parameters *N* and *p*=def1−(1−*π*)^*d*^. Note that *p* depends on the duration of the epoch (through *d*). In the next epoch there are *N*−*n*_1_ unregistered individuals in the population and conditionally on *n*_1_, the random variable *n*_2_ is distributed as a Binomial variable with parameters *N*−*n*_1_ and *p*. Continuing this argument it follows that

(1) $$n_{j}|n_{1},n_{2},\ldots n_{j-1}∼\text{Bin}\left(N-\overset{j-1}{\underset{i=1}{\sum }}n_{i},p\right).$$

The log-likelihood function of *N* and *p* given the data *n*_1_,*n*_2_,…,*n*_*M*_ is given by (e.g. [15])

(2) $$\begin{array}{ll} L_{M}= & \log(p)s_{M}+N\log(N)\\ & +\overset{M}{\underset{i=1}{\sum }}\left(N-\overset{i}{\underset{j=1}{\sum }}n_{j}\right)\log(1-p)\\ & -(N-s_{M})\log(N-s_{M})+C \end{array}$$

Here *s*_*M*_ denotes the sum of the observations:

$$s_{M}\overset{\text{def}}{=}\overset{M}{\underset{i=1}{\sum }}n_{j},$$

and *C* is represent terms that do not depend on *N* or *p*. To derive the likelihood equation (Eq. 2) the following standard approximation, valid for large *n*, was used:

n!≃nlog(n)−n.

Given the log-likelihood function (Eq. 2) we take as our estimate of population size the value of *N* that maximizes that expression (henceforth denoted *N*_*M* *L*_). Note that we simultaneously estimate the registration probability *p*, but since this is not the parameter of main interest, we will focus on the estimator of *N*.

For the case *M*=2 the maximum of the likelihood function is given by $N_{\text{ML}2}=n_{1}^{2}/(n_{1}-n_{2})$. However, in the general case, the maximum likelihood estimates are easiest found by maximizing Eq. 2 numerically, for example using Newton’s method. It should be noted that the ML estimator is not applicable for all samples. For the case *M*=2, for example, *n*_1_ must be greater than *n*_2_. General conditions for when the ML estimator is applicable have been derived before [18]. As we will show later, in cases where the ML estimator give good estimates of *N*, it is very unlikely to get a sample for which the ML estimator does not exist.

### Truncated poisson estimate of ***N***

Given a single source of data with possible multiple registrations per person there are alternative estimates of populations size that can be (and have been) used. The simplest of these estimates can be derived if one assumes that the sampled data follow a zero-truncated Poisson distribution (this is just a re-weighted standard Poisson distribution in which the zero bin is not observed). We note that if the probability *π* of being registered at each time point is independent of previous registrations, and there are *T* time points in total, then the number of registrations for each subject is distributed as a binomial variable with parameters *T* and *π*. Given reasonable values of these parameters, the distribution of the number of registrations will closely follow a Poisson distribution with parameter *λ*=def*Tπ* (e.g. [19]). Consider for example that observations are made daily for a year, then *T*=365, and if the probability of being registered at least once in a year is 0.3 then *π*≃0.001 which gives *λ*≃0.36. These are values for which the Poisson approximation of the Binomial distribution is very good and, consequently, a zero truncated Poisson distribution is a natural choice to model the distribution of the registrations.

To derive estimators from this distribution we may proceed as follows. With *λ* being the parameter in the Poisson distribution (the rate), the probability mass function of the untruncated distribution has the following form

$$f(k;\lambda )=\text{Pr}(X=k)\overset{\text{def}}{=}\frac{\lambda^{k}e^{-\lambda }}{k!}.$$

Here *X* denotes the random variable standing for the number of registrations made (for one person). Now, draw *N* independent samples, *X*_*i*_, *i*=1,2,…*N* following this distribution (here *N* is of course the (unknown) size of the population) and let *h*_*j*_ denote the count of cases where *X*_*i*_=*j* for *j*=1,2,…*T*. That is, *h*_1_ is the number of observed cases that was registered exactly once, *h*_2_ the number of cases observed exactly two times, etc. Note that in applications *h*_0_ is not observed; it represents the “hidden” part of the population. The *h*_*j*_s are random variables with the following expected values

$$\begin{array}{l} E(h_{0})=Ne^{-\lambda },\;E(h_{1})=\mathrm{N\lambda}e^{-\lambda },\\ E(h_{2})=\frac{N\lambda^{2}e^{-\lambda }}{2}. \end{array}$$

From this it follows that

(3) $$E(h_{0})=\frac{{\left(E(h_{1})\right)}^{2}}{2E(h_{2})}.$$

If we replace the expected values with the sample values we get the following simple estimator of *N*:

(4) $$N_{C}=\overset{T}{\underset{i=1}{\sum }}h_{i}+\frac{h_{1}^{2}}{2h_{2}}.$$

By the law of large numbers we know that *N*_*C*_ will be close to *N* when *N* is large. We will investigate the convergence further below. This estimator (*N*_*C*_) was first derived in [17], using a more general formalism and we will refer to *N*_*C*_ also as Chao’s estimator.

An alternative estimator can be obtained by first estimating *λ*, and then using that

$$N(1-e^{-\lambda })=E\left(\overset{T}{\underset{i=1}{\sum }}h_{i}\right).$$

The parameter *λ* can, for example, be estimated as

$$\lambda_{e}=\frac{2h_{2}}{h_{1}},$$

which leads to the following estimator of *N*

$$N_{Z}=\frac{\overset{T}{\underset{i=1}{\sum }}h_{i}}{1-e^{-2\frac{h_{2}}{h_{1}}}}.$$

This estimate was derived in [10] using a different approach.

### Monte Carlo simulations

To evaluate the performance of the estimators we performed Monte Carlo simulations. In these simulations we generated data for 512 ‘time steps’ (e.g. days) where each of *N* individuals has a probability *p* of being registered. Simulations where run for different values of *N* and different values of *p* (described in the Results). All simulation results were based on 5000 realizations. For the ML estimator the maximum of the likelihood (Eq. 2) was found numerically (for *M*>2) using Newtons method. In some cases the method did not converge, something that often was due to a failure of automatically specifying initial conditions within the region of convergence. In such cases a small change to the initial conditions is often sufficient for convergence. Since there is an explicit expression for the case *M*=2, the corresponding estimate is a good starting point for the algorithm. Although it is possible (in particular for small samples) to get data for which the ML estimator does not exists [18], for our purposes problems of convergence were very rare in all cases where the estimator will be useful in practice (see Results). An indication that a sample is not suited for the ML estimator is if *n*_1_≤*n*_2_ (for the case of *M*=2), and if so other methods of estimation must be used.

The ML estimator was derived assuming that all the individuals in the population have the same registration probability, something that translates to *p* in the simulations is the same for individuals. To model the effect of heterogeneity in the target population we run simulations where we allowed *p* to vary from individual to individual. In particular, the *p*s were randomly sampled from a normal distribution with a fixed mean value. The parameter used to control population heterogeneity was the standard deviation of the normal distribution. This way the effect of increasing population heterogeneity can be studied systematically from a constant *p* (zero standard deviation) to *p*s that vary a lot between individuals (large standard deviation). We also run simulations where we sampled *p*s from uniform distributions but the results were similar to those obtained using a normal distribution and are therefore not shown.

### Empirical data

Apart from the evaluation done on simulated data, we also wanted to use the suggested method on ‘real’ data. Here it was deemed important to use a data set where *N* could be assumed known, since then we can evaluate how close to the true value the different estimates are. We approached this by using as our target ‘population’ the accidentally deceased opiate misusers (fatal overdoses) in Sweden during 2006–2011. Most such cases occur outside of hospitals and the causes of death are therefore investigated in detailed forensic autopsies (including a toxicological analysis). We can therefore use the central Swedish Causes of Death registry held by The National Board of Health and Welfare (Socialstyrelsen) to count these cases and get a reasonably precise value of *N*.

To identify the deceased persons making up the target population we proceeded as follows. We selected all persons between 23 and 60 years of age at the time of death who were listed in the causes of death registry with heroin or methadone poisoning (ICD-10 codes T40.1 and T40.3) as contributing cause of death. This choice of substances should be obvious. However, there are also a substantial number of cases with morphine poisoning (T40.2) but these were not included as they were judged to constitute a mixed group of individuals, where only some are opiate misusers in the sense relevant here. The upper age limit was to minimize the number of cases where methadone had been medically prescribed and used in pain management therapy. The lower age limit was to enable a search of five year medical history for all individuals (we are tacitly assuming that most younger persons in this population had started their opiate misuse by the time they were 18 years old). We furthermore excluded all cases that were judged to be suicides. After applying these criteria there were 486 persons remaining who can be assumed to have misused opiates on a regular basis and these 486 will constitute our target population. To evaluate the population size estimators we will now see how well we can estimate the size of this population from another set of data. For this we will use health care data as described next.

For each of the *N*=486 individuals in the population we extracted information on previous contacts with the Swedish health care system by using the National Patient Registry (held by The National Board of Health and Welfare). This registry contains all instances of inpatient care in Sweden with good coverage and contains the main diagnosis (classified according to the WHO ICD-10 classification system) and time points for care. For the target population we extracted for all occurrences of inpatient care under diagnoses indicative of substance misuse. In particular, we used the following ICD-10 diagnoses: F11-F16, F18, and F19 and restricted the search to five years immediately preceding the time of death. During these time intervals 262 of the 485 persons (54%) had been receiving care under these diagnoses. We note that this might be taken as support for the assumption that the 485 persons constitute a relatively homogeneous population with respect to substance misuse. There were a total of 1165 hospital records from these 262 persons and most individuals had more than one care occasion. These health-care records will be used below to estimate *N*.

Since the individuals were all deceased at the time of project initiation, ethics committee approval was not necessary according to the regional research ethics board (Etikprövningsnä mnden i Stockholm).

## Results

This section starts with a study of the ML estimator and its performance under variations of total population size and fraction of the populations actually sampled. We then go on to study the effect of introducing variability (from unit to unit) in the registration probability (i.e. we make the population heterogeneous) and then the effect of the number of epochs that the sampling interval is divided into. Subsequently we will study Chao’s estimator and describe when it will fail to provide good estimates. Finally we apply the estimators to a real data set.

### Variability and bias of the ML estimator

Ideally, an estimator of population size should always give the right answer. However, since the estimator is computed from data (assumed to be random) it will both be variable (i.e. will give different results for different realizations) and it might have a bias (i.e. the expected value of the estimator might differ from the true value). In this section we study how the variance and bias of the estimator depend on total population size (*N*) and the fraction of *N* that is sampled (observed).

**Figure 2:**
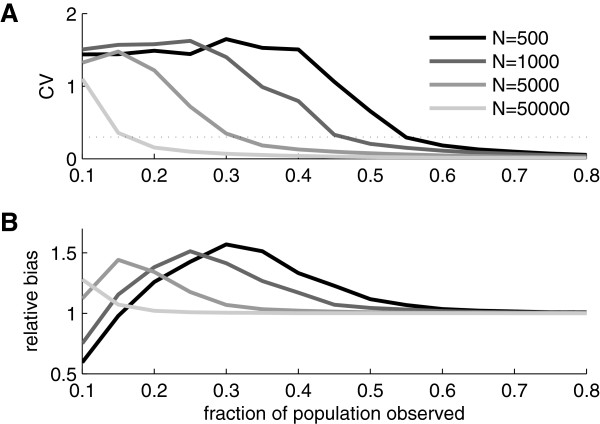
**Variability and bias of the estimator. A)** Coefficient of variation (CV) of population size estimator as estimated from simulated data for four different population sizes. The dotted line indicates a CV of 0.3 which might represent an acceptable level of variability in practice. **B)** Bias of population size estimator as estimated from simulated data. The curves show sample mean divided by the nominal population size (relative bias) for four different population sizes.

To study the variability and bias of the estimator we made Monte Carlo simulations for *N*=500,1000,5000, and 50000 and for each *N* we varied the fraction of observed units from 0.1 to 0.8 in steps of 0.05. To characterize the variability we used the estimated coefficient of variation (CV) (sample standard deviation divided by sample mean). Results are shown in Figure 2A. For all *N*s the CV starts with a high value and then decreases as the fraction sampled increases.

To investigate the bias of the estimator we divided the sample means from the simulations with their corresponding theoretical value (i.e. with the corresponding *N*s). The resulting measure will be referred to as relative bias. Note that a relative bias of one means that the estimator is unbiased. Figure 2B shows the relative bias for the parameter values investigated. For sufficiently large fraction of sampled units, the bias goes to zero independently of population size *N*. However, for smaller fractions sampled, the bias can be substantial and for the two smallest populations (*N*=500 and *N*=1000) the bias is a non-monotonous function of sample size; it starts of with a substantial negative bias that later becomes positive before going to zero (relative bias going to one).

If we consider these bias and variance results together they show that whether the ML estimator is useful or not depends strongly on both *N* and the sample size. For example, if the true *N* is in the order of 50000 then already with a sampled fraction of about 15% (i.e. a sample size of 7500) very good estimates are typically obtained. If, on the other hand, the true *N* is around 500 the sampled fraction should not be less than 55% (sample size of 275) for the estimator to be reasonably accurate.

### Population heterogeneity

So far we have assumed that the population is homogeneous with respect to the probability of being registered in each time interval (at least before being registered the first time). This translates to a constant *p* for all ‘subjects’ in the simulations. In the real world this assumption is obviously unrealistic and in this section we study the effects of relaxing it (see Methods).

**Figure 3:**
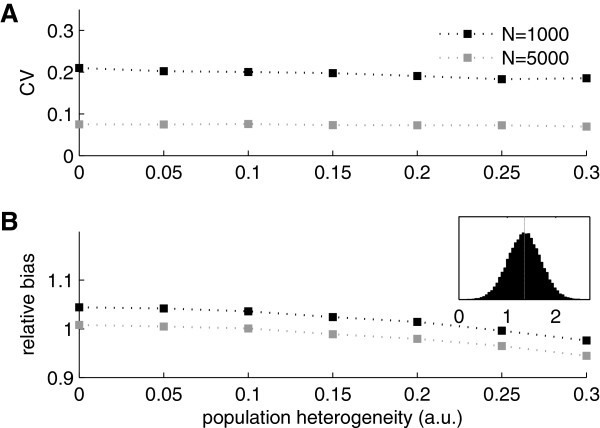
**Effects of heterogeneity on population size estimator. A)** Coefficient of variation of population size estimator as estimated from simulated data as a function of population heterogeneity for two different population sizes. The registration rate parameter *p* was randomly varied from unit to unit according to a normal distribution. The population heterogeneity was taken as the standard deviation of this normal distribution (x-axis). **B)** Bias of population size estimator as estimated from simulated data for different levels of population heterogeneity. The inset shows a histogram illustrating the distribution of rate parameter *p* corresponding to a heterogeneity of 0.25 (the *p*s have been scaled by 1000 to improve readability). All simulations in this figure were run with a sample size of 50% of the total population size.

Figure 3 shows the effect of making the units different with respect to the probability of being registered. Over the range studied, the heterogeneity does not influence the performance of the estimator greatly. Indeed, as shown in the inset in Figure 3B, the variability in registration probability between individuals can be substantial without leading to detrimental performance. Of course, if the variability in *p* (between units) become sufficiently large the performance will eventually break down. In particular, when many units start having zero probability of being registered the estimator will be biased.

### Choosing ***M***

In applications of the capture-and-remove method in population biology the number of observation occasions (captures) might often be determined by the design of the study. In epidemiology, on the other hand, we typically have a certain freedom to choose the number of ‘observation occasions’ (epochs, denoted *M* herein). Indeed, we might have access to daily registrations for a year or more and it is therefore of interest to see how dividing the total time interval into epochs of different length will influence the performance of the estimator. We investigated this by running Monte Carlo simulations for different values of *M*. We used *M*=2,4,8,16,32, and since the total time interval consisted in 512 observation units (‘days’) we used exactly the same data in all these five cases. Note that the case *M*=2 is special in that a closed-form expression exists whereas for *M*>2 the ML estimate has to be found iteratively.

**Figure 4:**
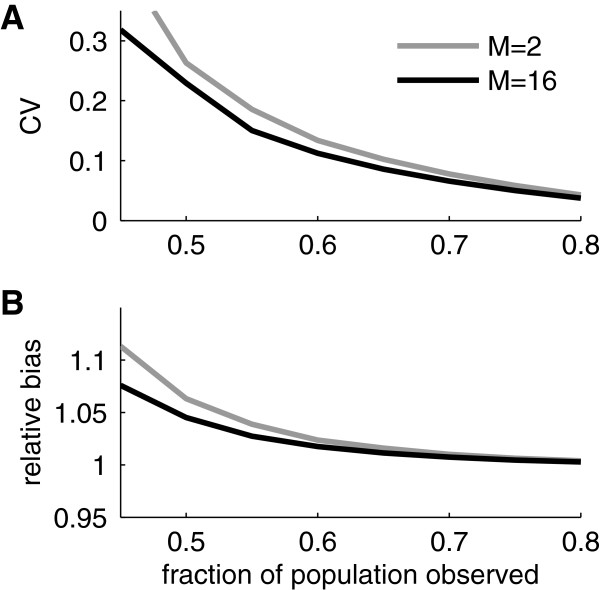
**Performance as a function of *M. *A)** Coefficient of variation of two ML estimators corresponding to *M*=2 and *M*=16 as determined from simulated data for a population if *N*=1000. **B)** Relative bias of the two ML estimators as estimated from simulated data.

We found that when the fraction of the total population sampled was large enough to give a relative variability (coefficient of variation) of 0.3 or smaller then the estimates obtained for *M*≥8 were virtually identical (correlation coefficients between estimates obtained for different values of *M* were ≥0.98). However, compared to the case of *M*=2, estimates differed and even if the correlation coefficient was relatively high (around 0.8) the estimates with *M*=2 had slightly larger coefficients of variation and a larger bias. Indeed, Figure 4 shows the simulation results for *N*=1000 and compares the performance of the estimator with *M*=2 to that with *M*=16. It is clear that the size of the effects of *M* are rather small but that in general an estimator with *M*≥8 should be used.

For smaller fractions of the population size the different estimators have less regular behavior, and correlation between different estimates might be as low as 0.35. Varying *M* and comparing the resulting estimates might therefore constitute a check that a sufficient fraction of the total population has been sampled.

### Truncated poisson estimation

A simple alternative to the estimator we are suggesting when having data from one source is to assume that the samples come from a zero-truncated distribution and use all, or parts of the data to estimate the unobserved zero bin (e.g. [10]). For the type of data considered here a powerful and often used estimator goes under the name of Chao’s estimator. For the truncated Poisson distribution this estimator is easy to derive (Methods) but it was originally derived in a more general setting [17]. In this section we investigate the performance of this estimator (*N*_*C*_) and will see how it fails when we introduce simple dependencies between the probabilities of beingregistered.

We note that there are alternative ‘truncated Poisson’ estimators and one that has been applied was suggested in [10] and is also derived in the Methods. We made extensive tests using also this estimator and on our simulated data it behaved very similar to *N*_*C*_ but had a slightly larger variance. We will therefore focus on *N*_*C*_ in thefollowing.

#### Variability and bias of Chao’s estimator

**Figure 5:**
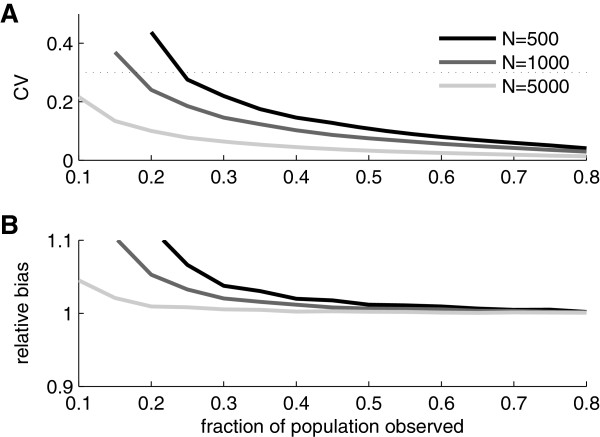
**Variability and bias of the truncated Poisson estimator. A)** Coefficient of variation of Chao’s estimator as determined from simulated data for three different population sizes. **B)** Bias of Chao’s estimator as estimated from simulated data. The curves show sample mean divided by the nominal population size (relative bias) for three different population sizes.

The variability and bias of Chao’s estimator was determined analogously to that of the ML estimator. The results are shown in Figure 5. It is clear that when the Poisson assumption is fulfilled, Chao’s estimator outperforms the ML estimator. This is in particular the case when only a small fraction of the population is sampled. For the case *N*=500, for example, Chao’s estimator gives reasonably reliable estimates already when the sample size is around 125. However, when the assumptions are violated, Chao’s estimator can have a substantial bias, even when the fraction sampled islarge.

#### Systematic error in Chao’s estimator

One crucial assumption for any estimator relying on a truncated Poisson distribution is that the probability of being registered at any time is independent of previous registrations. In epidemiological data in general, and in health care records in particular, it is unlikely that this assumption holds. We will investigate this in an example data set below.

**Figure 6:**
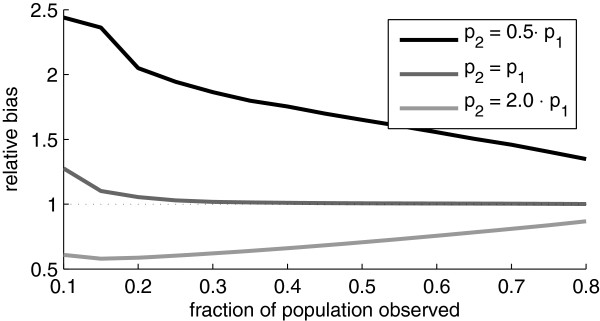
**Bias of the truncated Poisson estimator when *p***_***2 ***_***≠ p***_***1***_**.** Relative bias (sample mean divided by nominal population size) of Chao’s estimator as estimated from simulated data when the probability of being registered more than one time was varied. The nominal population size was 1000.

Here we will study the behavior of Chao’s estimator when the probability of being registered two or more times is different from the probability of being registered once. In the Monte Carlo simulations we started with all first-time registration probabilities (*p*_1_) equal and then once a unit was registered we changed (or not) the future registration probability *p*_2_. The effect of this will be that the distribution of the registrations no longer follow a Poisson distribution. As shown in Figure 6 the effect on Chao’s estimate of violating the Poisson assumption can be substantial. When a registered unit is less likely to become registered again, Chao’s estimator has a large positive bias, and this even for large sample sizes. When the probability of being registered more than once is larger than the probability of first registration, the estimator has a strong negative bias.

The reason for this large bias can be seen from the following considerations. Assume that that *p*_2_=*p*_1_/*k*, where *k* is an integer parameter. Then if *N* is large and *p*_1_ is small

$$\frac{E{\left(h_{1}\right)}^{2}}{E(h_{2})}≃\text{kN},$$

which implies that Chao’s estimator will have an arbitrarily large positive bias.

### Application to real data

We next apply *N*_*M* *L*_ and *N*_*C*_ to a real data set. We wanted to use the estimators on a population where we already knew (or at least had a very good approximation of) the size from other sources. This can be viewed as an attempt to validate the methods using real data; only by knowing the true size can we determine which of the methods that give the best estimate. As explained in Methods, the cases of fatal opiate overdoses in Sweden from 2006 to 2011 is a candidate for such a population. We took the total size of this population to be number of deceased as reported by The National Board of Health and Welfare. It totaled 486 persons. Now we will now see how well we can ‘estimate’ this number from the available health care records from the deceased persons. There were 262 of these individuals that had received inpatient care under the diagnoses of interest (see Methods) during this time.

#### Application of ***N***_***ML***_

To estimate the number of cases using the ML estimator we need to keep track of the date of the first care instance for each of the 262 persons. Then we divide the five years into a number (*M*) of epochs. As shown above, given this sample size we should expect about the same performance for *M*=2 as for higher values of *M*. When applied to the data we got the following estimate for *M*=2: 462, which is in reasonable agreement with the true *N*. Increasing *M* had relatively small effects on the estimate: (*N*_*M* *L*:5_=461; *N*_*M* *L*:10_=431; *N*_*M* *L*:20_=435).

#### Application of ***N***_***C***_

To use *N*_*C*_ to estimate the population size we used all the 1156 observations from the health care registry. We count how many persons that received care 1 and 2 times and then use the formula stated in the Methods (Eq. 4). Doing this showed that 75 persons had been in care one time only and 55 persons had been in care twice. This lead to an estimate of *N*_*C*_=313, i.e. a substantial underestimate.

Next we try to understand the reason for this underestimate. For Poisson data we know that the ratio of zero to one observations should equal half the ratio of one to two observations or in other words that

$$\frac{h_{0}}{h_{1}}=\frac{h_{1}}{2h_{2}}.$$

For the data from the opiate overdose cases we get that $\frac{h_{0}}{h_{1}}=2.49$ and $\frac{h_{1}}{2h_{2}}=0.68$ which can be interpreted that once a person has been registered (in care for substance misuse) the probability of being registered again is substantially increased.

## Discussion

We have studied the performance of a novel (in this context) population size estimator and delimited the situations when it might be useful. The method is tailored to epidemiological data and can, for example, be used to track the prevalence of drug misuse. It relies on two assumptions: first that the population is closed, i.e. that individuals do not enter or exit the population during the time of study. Second, that the probability of being registered at least once is constant among the members of the population. We showed that reasonable violations of the second assumption does not invalidate the estimator (Figure 3).

The first assumption is unlikely to hold exactly in real cases but can be partly circumvented by restricting the analysis to a short period of time. Assume for example that it is of interest to estimate the number of problem drug users in a region. Now, clearly this population is not closed, someone can be a problem drug user for a given time period and then quit, whereas new individuals can enter at any time point. Still, if we restrict the time horizon to a few years the population flux presumably becomes much smaller. Also, whether the closed population assumption is reasonable or not depend on the temporal stability of the feature of interest, and on the total size of the population. If the population size is small and the conditions for being in the population are variable, it might be better to use other methods of estimation.

We also studied a different population size estimator (Chao’s estimator) and under the assumption that the registrations follow a zero-truncated Poisson distribution this method outperforms the ML estimator (Figure 5). In other words, when there are good reasons to believe that the registrations follow the Poisson assumption, one should use Chao’s estimator. We also note that in this case the performance of this estimator was slightly superior (smaller variance) to an alternative estimate also derived from truncated Poisson data (Zelterman’s estimator). However, and importantly, we also showed that Chao’s estimator can have a substantial bias (systematic error) when the Poisson assumption is violated (Figure 6). In the real data set we analyzed, Chao’s estimate was a substantial underestimation of the true value. This is a strong indication of that having had contact with the health care system changes the propensity for future contacts. As this could certainly be a general feature of similar data it questions the use of Chao’s estimator in suchsituations.

In practice it might be difficult to investigate if the probability of being registered is dependent on previous registrations or not. However, an indication of this can be obtained by analyzing parts of the data separately. Assume that registrations are followed over a period of time. Since estimates obtained by Chao’s estimator should not strongly depend on the duration of the time period used, similar estimates should be obtained if the first half of the time period is used compared to if the whole time period is used. Of course, as the sample size increase the estimates become more precise, but large dependencies on the time period is a clear indication of that truncated Poisson estimators might have a substantial bias and should preferably not be used.

There are generalizations of the zero-truncated distribution approach that might fare better under the perturbations we introduced and that are better suited to the type of data we have in mind. More generally, one can divide the observation interval into epochs (as we have done) and model observations in each epoch and overlaps between the different epochs, for example using generalized linear models. This would be similar in spirit to the standard capture-recapture approach [6]. However, such approaches rely on more knowledge of the data generating process, knowledge that often might not be available. Alternatively, more parameters must be estimated from data and it is therefore unclear what would be gained in terms of power. We therefore believe that it is important to use estimators that depend on as few assumptions as possible, such as the ML estimator we have studied here. Of course, in practice, and depending on the data available, it typically would make sense to use different estimators of population size. If results differ wildly in unexplainable ways one should perhaps use completely different methods to estimate the population size.

We end with some practical notes. The input to the ML estimator should be the time point of first registration (e.g. care under a certain diagnosis). But since it will often be necessary to limit the time period used in the analysis, it might be important to make sure that the registrations in the first epoch actually reflect the first time of registration (and not the first time of registration *within the time window of the study*). For example, in the data we analyzed here we used registrations in a time interval of five years preceding time of death. However, any registration during the first epoch could in fact be the *n*-th registration, where the previous *n*−1 fell outside the five year interval. One way of circumventing this is to not use the data from the first half year or so. That way we can make sure that the first registrations used in the estimation are at least not part of a high frequency sequence of visits. In fact, when we excluded the first six month of data in our application of the method, the ML estimator gave a slightly more accurate result (e.g. *N*_*M* *L*20_=509).

Another practical issue relates to the choice of *M*. Given data acquired on a daily basis it is up to the user to choose a reasonable value for this parameter. When the observed sample constitute a substantial fraction of the total population the estimate does depend strongly on *M* and we showed that a value of *M* around 8 would be enough to get a close to optimal performance. However, it is still instructive to try different values of this parameter since a substantial dependence on *M* indicates that the estimated values should not be trusted.

## Conclusion

The population size estimator we have introduce here complements existing methods and is less sensitive to certain types of dependencies that might be typical in epidemiological data. When given a single source of data it should therefore be considered the method of choice.

## Abbreviations

ML: maximum likelihood; WHO: World Health Organization; ICD-10: International Statistical Classification of Diseases and Related Health Problems, 10th Revision.

## Competing interests

The authors declare that they have no competing interests.

## Authors’ contributions

AL conceived, derived, and implemented the ML estimator; designed and analyzed the Monte Carlo simulations and analyzed the real world data; wrote the paper. PW planned the research and edited the paper. Both authors read and approved the final manuscript.

## Pre-publication history

The pre-publication history for this paper can be accessed here:

http://www.biomedcentral.com/1471-2288/14/58/prepub
